# Critical role of right heart catheterization in the evaluation of the appropriateness of transcatheter repair of severe tricuspid valve regurgitation: a case report

**DOI:** 10.1093/ehjcr/ytag009

**Published:** 2026-01-09

**Authors:** Micha T Maeder, Philipp Baier, Sebastian Hasslacher, Hans Rickli, Philipp K Haager

**Affiliations:** Cardiology Department, Health Ostschweiz (HOCH) Kantonsspital St. Gallen, Rorschacherstrasse 95, St. Gallen CH-9007, Switzerland; Cardiology Department, Health Ostschweiz (HOCH) Kantonsspital St. Gallen, Rorschacherstrasse 95, St. Gallen CH-9007, Switzerland; Cardiology Department, Health Ostschweiz (HOCH) Kantonsspital St. Gallen, Rorschacherstrasse 95, St. Gallen CH-9007, Switzerland; Cardiology Department, Health Ostschweiz (HOCH) Kantonsspital St. Gallen, Rorschacherstrasse 95, St. Gallen CH-9007, Switzerland; Cardiology Department, Health Ostschweiz (HOCH) Kantonsspital St. Gallen, Rorschacherstrasse 95, St. Gallen CH-9007, Switzerland

**Keywords:** Tricuspid regurgitation, Percutaneous repair, Shunt, Heart failure with preserved ejection fraction, Right heart catheterization, Case report

## Abstract

**Background:**

Transcatheter tricuspid valve repair is an increasingly performed procedure, which improves quality of life in selected patients with severe tricuspid regurgitation (TR). For the evaluation of the appropriateness of this procedure, identification of the exact mechanism(s) underlying TR is mandatory. Guidelines recommend right heart catheterization (RHC) as part of the work-up in these patients.

**Case summary:**

A 80-year-old man with shortness of breath, atrial fibrillation, and severe TR was referred for transcatheter repair. Echocardiography showed a preserved left ventricular ejection fraction, a dilated right ventricle with impaired function, severe functional TR, and a high probability of pulmonary hypertension (PH). Right heart catheterization revealed a mean pulmonary artery (PA) pressure of 34 mmHg and a mean PA wedge pressure (mPAWP) of 18 mmHg. The PA oxygen saturation was 79%, and an anomalous vessel draining into the superior vena cava with an oxygen saturation of 97% was found, which was identified as partial anomalous pulmonary venous return with a left-to-right shunt (ratio of pulmonary to systemic blood flow of 1.8:1.0). The increased pulmonary blood flow and a mildly elevated pulmonary vascular resistance of 2.3 Wood units resulted in an increased transpulmonary gradient, which added to an elevated mPAWP (heart failure with preserved ejection fraction) led to combined pre- and post-capillary PH.

**Discussion:**

This case highlights the importance of RHC in the evaluation of patients with severe TR. Here, TR was the result of right ventricular volume and pressure overload with very limited treatment options, and percutaneous repair was not appropriate.

Learning points

- Guidelines recommend right heart catheterization during the work-up of patients with severe tricuspid regurgitation evaluated for transcatheter repair.
- The present case highlights that right heart catheterization can identify previously unknown mechanisms of tricuspid regurgitation such as a congenital left-to-right shunt.
- Use of a combined non-invasive and invasive approach is essential to identify those who benefit from the procedure and even more importantly also those in whom it may cause harm.

## Introduction

Transcatheter tricuspid valve repair is an increasingly performed procedure, which improves quality of life in selected patients with severe tricuspid regurgitation (TR).^1^ Whereas primary TR (clear anatomical abnormality of the tricuspid apparatus) is relatively rare, the majority of TR cases are either related to a cardiac implantable electronic device or most frequently secondary (functional) due right ventricular (RV; ventricular TR) and/or right atrial (atrial TR) dilatation/dysfunction, which can have numerous reasons including primary RV disease (e.g. RV cardiomyopathy), atrial fibrillation (AF), or various forms of pulmonary hypertension (PH).^2,3^ Although TR itself can cause symptoms by congestion and forward failure,^4^ symptoms in patients with severe TR are often at least in part the consequence of the underlying cardiac and/or pulmonary disease rather than the result of TR *per se*.^3^ Therefore, a detailed non-invasive and invasive evaluation of the mechanisms of TR is essential before performing TR repair. In particular, current guidelines on the evaluation and management of patients with valve disease^2^ and PH^5^ recommend right heart catheterization (RHC) as part of the work-up to assess the presence, extent, and mechanism(s) of PH. Here we present a case, where we highlight the critical importance of RHC in this context.

## Summary figure

**Figure:**
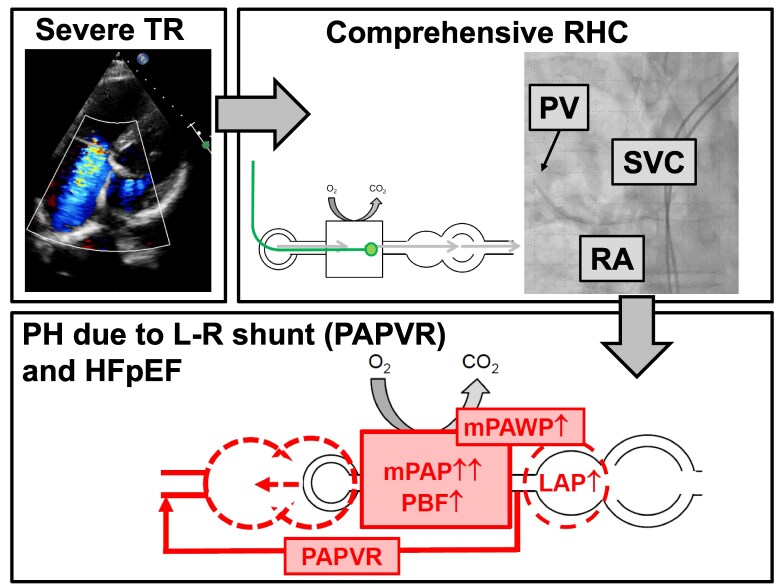
HFpEF, heart failure with preserved ejection fraction; L-R, left-to-right; PAPVR, partial anomalous pulmonary venous return; PV, pulmonary vein; RA, right atrium; RHC, right heart catheterization; SVC, superior vena cava; TR, tricuspid regurgitation.

## Case presentation

**Figure 1:**
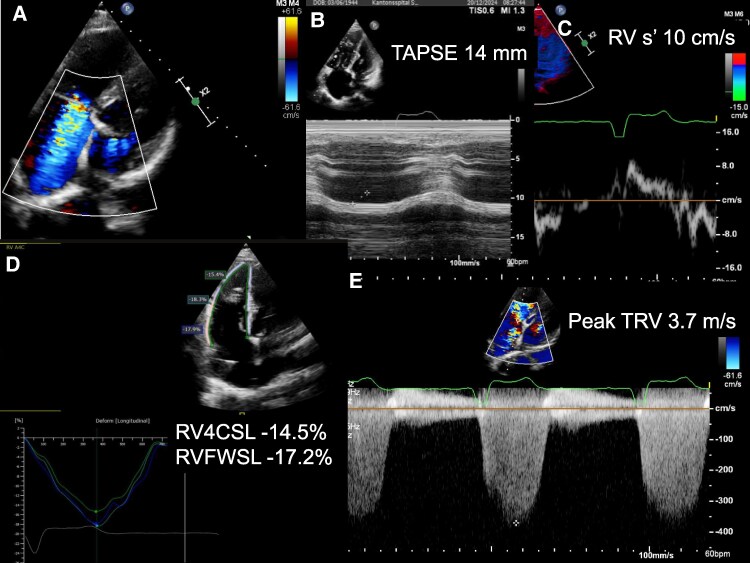
Transthoracic echocardiography. (*A*) Atypical four-chamber view showing severe tricuspid regurgitation. (*B*) M-mode showing reduced tricuspid annular plane systolic excursion (14 mm). (*C*) Pulsed wave tissue Doppler showing borderline right ventricular S’ (10 cm/s). (*D*) Speckle tracking showing reduced right ventricular four-chamber longitudinal strain and reduced right ventricular free wall longitudinal strain. (*E*) Continuous wave Doppler showing increased peak tricuspid regurgitant velocity (3.7 m/s) suggestive of a high probability of pulmonary hypertension.

**Figure 2:**
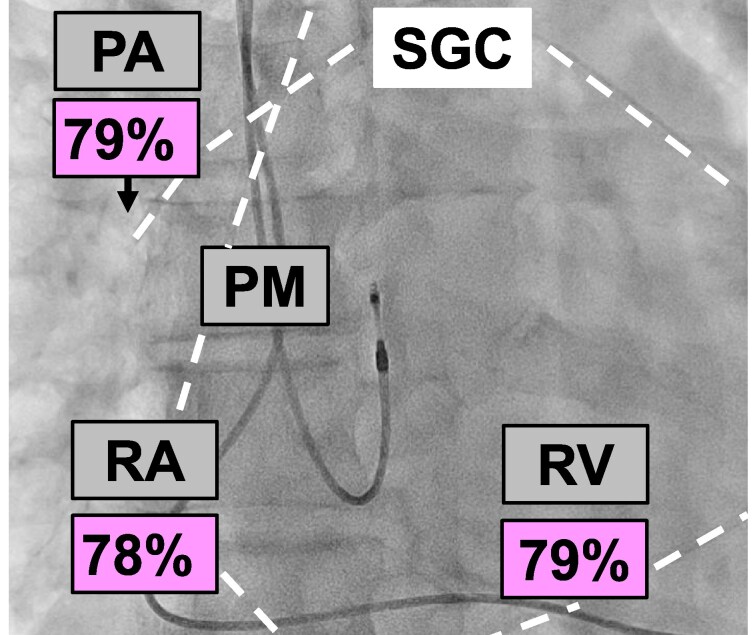
Right heart catheterization under fluoroscopy. Oxygen saturations in the pulmonary artery, right ventricle, and right atrium are indicated. The arrow points to the tip of the Swan–Ganz catheter (course highlighted by the white dashed line) in the right pulmonary artery. PM, pacemaker (double chamber pacemaker with leads in the right ventricle and right atrium).

**Figure 3:**
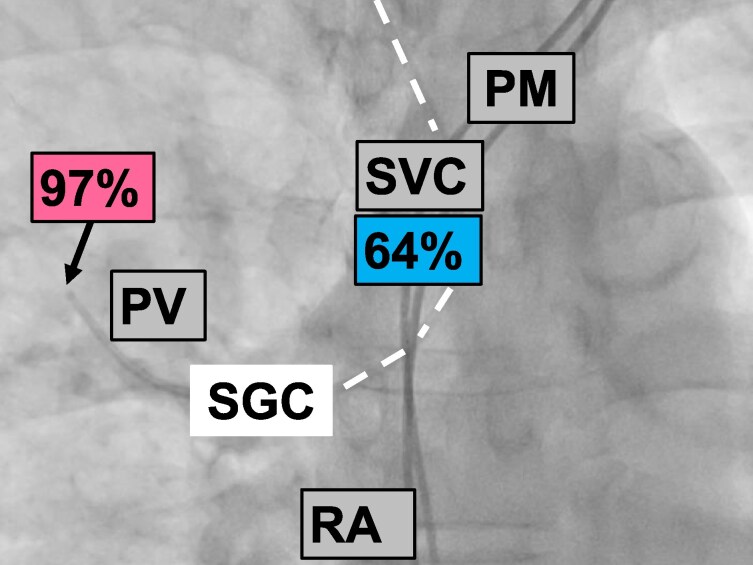
Right heart catherization under fluoroscopy. Oxygen saturations in the anomalous pulmonary vein and superior vena cava are indicated. The arrow points to the tip of the Swan–Ganz catheter outside of the heart (intracardiac Swan–Ganz catheter course highlighted by the white dashed line) in the pulmonary vein, which drains into the superior vena cava. PM, pacemaker; RA, right atrium.

**Figure 4:**
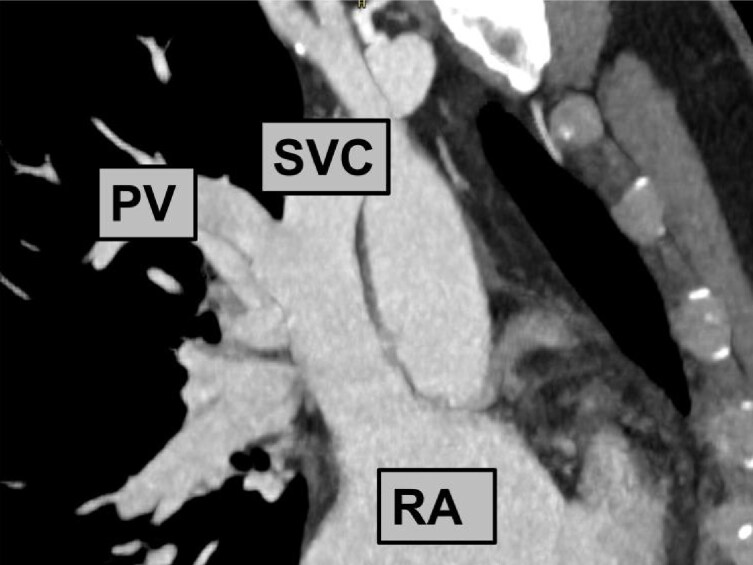
Computed tomography reconstruction of the anatomy (similar view as fluoroscopy in *Figure 3*). PV, pulmonary vein; RA, right atrium; SVC, superior vena cava.

**Figure 5:**
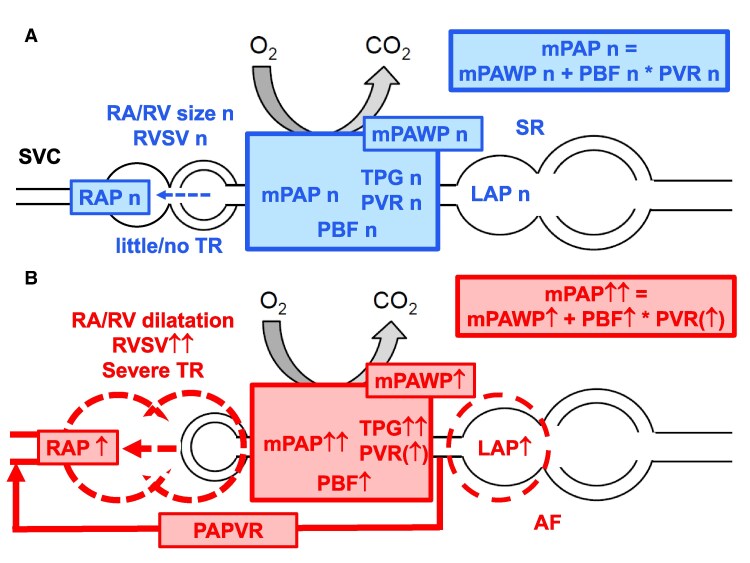
Schematic representation of the pathophysiology (simplified; the interaction between right and left ventricle is not shown). (*A*) Normal situation. (*B*) Present case (for detailed discussion, see text). AF, atrial fibrillation; LAP, left atrial pressure; mPAP, mean pulmonary artery pressure; mPAWP, pulmonary artery wedge pressure; PAPVR, partial anomalous pulmonary venous return; PBF, pulmonary blood flow; PVR, pulmonary vascular resistance; RA, right atrium; RAP, right atrial pressure; RV, right ventricle; RVSV, right ventricular stroke volume; SR, sinus rhythm; SVC, superior vena cava; TPG, transpulmonary gradient; TR, tricuspid regurgitation; *n*, indicates normal; (↑), borderline; ↑, elevated; ↑↑, substantially elevated.

A 80-year-old male suffering from shortness of breath [New York Heart Association (NYHA) Class III] was referred for the evaluation of percutaneous tricuspid valve repair. He had permanent AF and a pacemaker for recurrent syncope. Echocardiography showed a left ventricular (LV) ejection fraction of 55%, dilated atria, mild mitral regurgitation, and a dilated RV with impaired function (tricuspid annular plane systolic excursion 14 mm, RV S’ 10 cm/s, RV four-chamber longitudinal strain −14.5%, RV free wall longitudinal strain −17.2%; *Figure 1*; additional echocardiography data in Supplementary material online, *Table S1*). There were severe atrial functional TR (effective regurgitant orifice area 0.46 cm^2^, regurgitant volume 60 mL; see Supplementary material online, *Figure S1* and *Videos S1 and S2*; additional data in Supplementary material online, *Table S1*) and a high probability of significant PH (peak tricuspid regurgitant velocity 3.7 m/s, several indirect signs including RV dilatation, a D-shape of the LV, and a right atrial area >18 mm^2^) (*Figure 1*). There was no evidence of leaflet impingement by the pacemaker leads as cause of TR (see Supplementary material online, *Figure S2* and *Video S3*). Right heart catheterization revealed a mean right atrial pressure of 10 mmHg, a systolic/diastolic/mean pulmonary artery (PA) pressure of 74/13/34 mmHg, a mean PA wedge pressure (mPAWP) of 18 mmHg, and thus a transpulmonary gradient of 16 mmHg. The oxygen saturation in the PA was 79%, and the oxygen run revealed identical saturations in the RV and the right atrium (*Figure 2*). During the first attempt to advance the Swan–Ganz catheter from the superior vena cava (SVC) into the right atrium, an anomalous vessel was intubated, which had an oxygen saturation of 97%, while the oxygen saturation in the SVC was 64% (*Figure 3*). Thus, the patient had partial anomalous pulmonary venous return (PAPVR; one right pulmonary vein draining into the SVC), which was confirmed by computed tomography (*Figure 4*). A left-to-right shunt with a pulmonary blood flow of 7.1 L/min and a ratio of pulmonary to systemic blood flow of 1.8:1.0 was calculated. The resulting pulmonary vascular resistance (PVR) was 2.3 Wood units (WU). Given the limitations of the indirect Fick method for cardiac output assessment, cardiac magnetic resonance (CMR) imaging was performed revealing an identical shunt size of 1.8:1.0 but a slight lower pulmonary blood flow of 6.5 L/min (RV stroke volume index: 61 mL/m^2^, LV stroke volume index: 30 mL/m^2^; see Supplementary material online, *Figure S3*; additional CMR data in Supplementary material online, *Table S2*). Using the pressures from RHC and pulmonary blood flow from CMR, a very similar PVR of 2.5 WU was calculated. Thus, the increased pulmonary blood flow and the mildly elevated PVR resulted in a substantially increased transpulmonary gradient, which added to an elevated mPAWP [heart failure with preserved ejection fraction (HFpEF)] led to combined pre- and post-capillary PH (*Figure 5*). The patient was treated medically with oral anticoagulation, a beta-blocker, a loop diuretic, and a sodium–glucose cotransporter 2 inhibitor. A decision against a tricuspid valve intervention was made. Six months later, the patient was in NYHA Class II (see Supplementary material online, *Figure S4*).

## Discussion

This case highlights the importance of combined non-invasive and invasive imaging in patients with severe TR before a decision regarding percutaneous tricuspid repair is made. This patient had a clearly severe TR, which was the result of substantial RV volume (shunt plus TR) and pressure overload (PH). Pulmonary hypertension in the context of HFpEF can present with an ‘RV phenotype’ if there is not only isolated post-capillary PH (i.e. left atrial hypertension but no pulmonary vascular disease) but combined pre- and post-capillary PH (i.e. left atrial hypertension plus secondary pulmonary vascular involvement).^6^ This patient had an elevated mPAWP in the context of HFpEF with permanent AF. The presence of AF in a patient with evidence of PH always should lead to the consideration of a post-capillary component.^5^ However, there was a surprising second mechanism of PH, i.e. the left-to-right shunt due to PAPVR. This condition had been unknown prior to the present assessment, and it was very important that the diagnostic opportunity during RHC was not missed. Diagnostic clues included the saturation in the PA, which was much higher than expected for a patient with heart failure and low output, and the recognition of the abnormal structure draining into the SVC as a pulmonary vein by careful intubation and measurement of the oxygen saturation. Although this shunt had been present for more than 80 years and had caused increased pulmonary blood flow, there was no substantial pulmonary vascular disease. The PVR was only very mildly increased (2.3–2.5 WU depending on the method of pulmonary blood flow assessment; cut-off for abnormal: 2 WU.^5^) Given that treatment with pulmonary arterial hypertension (PAH)–specific drugs is an option for patients with unoperated left-to-right shunts and substantially elevated PVR and can even be considered in patients with left heart disease and post-capillary PH with very high PVR (>5 WU),^5^ meticulous assessment of PVR was essential. The PVR calculation critically depends on the measurement of the pulmonary blood flow, and the use of the indirect Fick method is limited by the fact that oxygen consumption is not directly measured but estimated based on nomograms.^7^ Therefore, measurement of pulmonary blood flow by a second method was important and we were able to show that pulmonary blood flow by CMR and RHC was essentially identical and measurements were therefore most likely reliable. Surgical correction of this type of shunt is not a simple operation and operation of PAPVR consisting of one single pulmonary vein is rarely appropriate^8^ and this is particularly true for a more than 80 years old patient. On the other hand, the response of the RV to tricuspid repair is unpredictable because patients with this specific pathophysiology were not included in studies. Just trying to shrink the effective regurgitant orifice area will not address the complex underlying pathophysiology (*Figure 5*). In general, the presence of PH predicts a poor prognosis after tricuspid valve repair,^9,10^ and the remodelling potential of the RV is limited by the persisting volume overload by the shunt and the pressure overload by the combination of an elevated mPAWP and an elevated transpulmonary gradient. Notably, a high transpulmonary gradient has also been identified as a predictor of poor prognosis after tricuspid repair.^10^

Accordingly, this was a situation with very limited treatment options (no indication for surgery for this type of congenital shunt at the age of 80 years, no indication for pacemaker lead revision, and no indication for a PAH-specific medical therapy because the PVR was clearly below 5 WU), and percutaneous tricuspid repair was not appropriate. In accordance with current ESC guidelines,^2,5^ RHC was instrumental as a basis for appropriate treatment decisions.

## Lead author biography


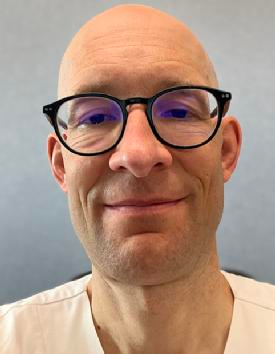


Micha T. Maeder, MD, PhD, is a consultant cardiologist at Health Care Ostschweiz (HOCH), St. Gallen, Switzerland. Apart from clinical training in Switzerland (Zürich, Basel, Bern, St. Gallen), he had undertaken a research fellowship at the Baker Heart and Diabetes Institute in Melbourne, Australia, and he has obtained a PhD degree at the University of Maastricht, Netherlands. His research interests include the pathophysiology of valve disease, heart failure with preserved ejection, and pulmonary hypertension. In clinical practice, he is involved in the non-invasive and invasive management of patients with coronary artery disease, valve disease, pulmonary hypertension, and heart failure.

## Supplementary Material

ytag009_Supplementary_Data

## Data Availability

The data underlying this article will be shared on reasonable request to the corresponding author.

## Supplementary material

**Consent:** Written informed consent was obtained from the patient for the publication of this case report, including all relevant clinical details and accompanying images. The patient was informed that all personal data would be anonymized to protect privacy, in accordance with COPE guidelines.
