# Supplementary material for: Critical role of right heart catheterization in the evaluation of the appropriateness of transcatheter repair of severe tricuspid valve regurgitation: a case report
Source: Eur Heart J Case Rep. 2026 Jan 9;10(1):ytag009. doi: 10.1093/ehjcr/ytag009 (PMC12826119; doi:10.1093/ehjcr/ytag009)
Supplement: ytag009_Supplementary_Data [file ytag009_supplementary_data.zip › R1_TR_supplementary_material_22.10.2025.pdf]

**Supplementary material for Case Report**

**“Critical role of right heart catheterization in the evaluation of the appropriateness of transcatheter repair of severe tricuspid valve regurgitation: a case report”**


**Page 2:** Supplementary Table S1

**Page 4:** Supplementary Table S2

**Page 5:** Supplementary Figure S1

**Page 6:** Supplementary Figure S2

**Page 7:** Supplementary Figure S3

**Page 8:** Supplementary Figure S4

**Page 9:** Legend to Videos S1-3

**Supplemental Table S1.** Detailed echocardiography parameters of right ventricular size and function, tricuspid valve and left ventricular function.

| | |
|---|---|
| **Right heart parameters** | |
| RV basal diameter (mm) | 46 |
| RV mid diameter (mm) | 36 |
| Indexed RV end-diastolic area ($cm^2/m^2$) | 12.9 |
| Tricuspid annular plane systolic excursion (mm) | 14 |
| RV peak systolic annular velocity (s’; cm/s) | 10.0 |
| RV fractional area contraction (%) | 43 |
| RV free wall longitudinal strain (%) | -17.2 |
| Right atrial area (4CH apical focused view; $cm^2$) | 25 |
| Inferior vena cava diameter (mm) | 24 |
| Peak tricuspid regurgitation velocity (m/s) | 3.7 |
| **Tricuspid valve parameters** | |
| Tricuspid annular diameter septo-lateral (3D; mm) | 44 |
| Tricuspid annular diameter antero-posterior (3D; mm) | 34 |
| Maximum leaflet gap size (mm) | 3 |
| Leaflet tenting height (mm) | 5 |
| Septal leaflet length (mm) | 12 |
| Effective regurgitant orifice area (PISA; $cm^2$) | 0.43 |
| Regurgitant volume (PISA; ml) | 60 |
| PISA radius at Aliasing velocity 0.35m/s (mm) | 8 |
| **Left heart parameters** | |
| LV ejection fraction (%) | 60 |
| Peak early mitral inflow velocity (E; m/s) | 1.27 |
| Peak early mitral annular velocity (e’; septal; cm/s) | 6.0 |

| | |
|---|---|
| Peak early mitral annular velocity (e’ lateral; cm/s) | 9.0 |
| E/e’ | 17 |
| Left atrial volume index (ml/m$^2$) | 42 |

**Supplemental Table S2.** Detailed cardiac magnetic resonance imaging (CMR) parameters.

| **CMR Parameters** | |
|---|---|
| Left ventricular (LV) ejection fraction (%) | 57 |
| LV end-diastolic volume (ml) | 88 |
| LV end-diastolic volume index (ml/m$^2$) | 52 |
| LV end-systolic volume (ml) | 38 |
| LV end-systolic volume index (ml/m$^2$) | 23 |
| LV stroke volume (total; ml) | 50 |
| LV stroke volume index (total; ml/m$^2$) | 30 |
| Forward flow (aorta; ml) | 44 |
| Backflow (aorta; ml) | 2 |
| Effective stroke volume (aorta; ml) | 42 |
| Right ventricular RV) ejection fraction (%) | 52 |
| RV end-diastolic volume (ml) | 167 |
| RV end-diastolic volume index (ml/m$^2$) | 98 |
| RV end-systolic volume (ml) | 64 |
| RV end-systolic volume index (ml/m$^2$) | 38 |
| RV stroke volume (total; ml) | 103 |
| RV stroke volume index (total; ml/m$^2$) | 61 |
| Forward flow (pulmonary artery; ml) | 7 |
| Backflow (pulmonary artery; ml) | 0 |
| Effective RV stroke volume (pulmonary artery; ml) | 79 |
| Tricuspid regurgitant volume (ml) | 24 |
| Tricuspid regurgitant fraction (%) | 30 |
| Shunt volume (ml) | 36 |
| Pulmonary to systemic flow ratio (Qp:Qs) | 1.8 |

**Supplemental Figure S1.** Multiplanar transgastric short axix view demonstrates a coaptation gap of 3mm in the central and mid regions of the anterior-septal coaptation line with mild curling of the septal leaflet (H) resulting in a centrally directed regurgitation jet (I).

A = Anterior Leaflet, S = Septal Leaflet, P = Posterior Leaflet

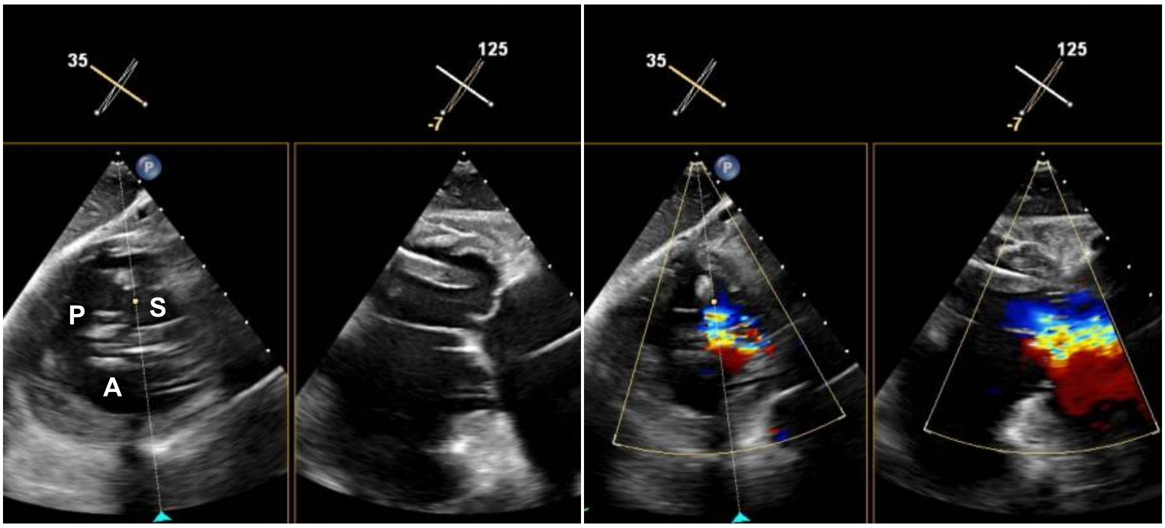

**Supplemental Figure S2.** Three-dimensional (3D) zoomed image from mid-esophageal window of the tricuspid valve (atrial perspective) in systole (F) and diastole (G) demonstrates pacemaker (PM) lead position in the posterior-septal commissure without leaflet impingement. A = Anterior Leaflet, S = Septal Leaflet, P = Posterior Leaflet, IAS = Interatrial Septum, AV = Aortic Valve.

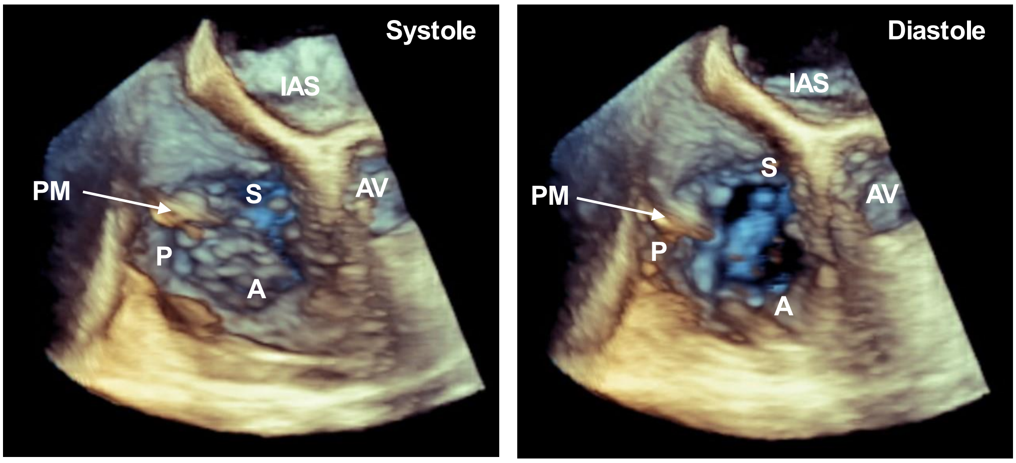

**Supplemental Figure S3.** Cardiac magnetic resonance imaging derived aortic and pulmonary artery blood flow.

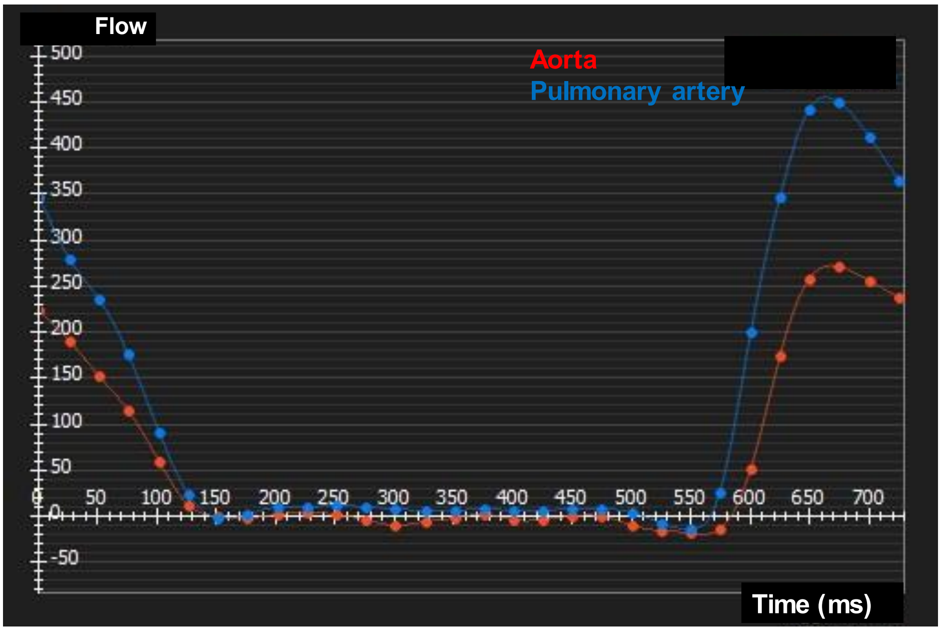

**Supplemental Figure S4.** Timeline of events.

NYHA = New York Heart Association, PAPVR = partial anomalous pulmonary venous return, PH = pulmonary hypertension, RV = right ventricle

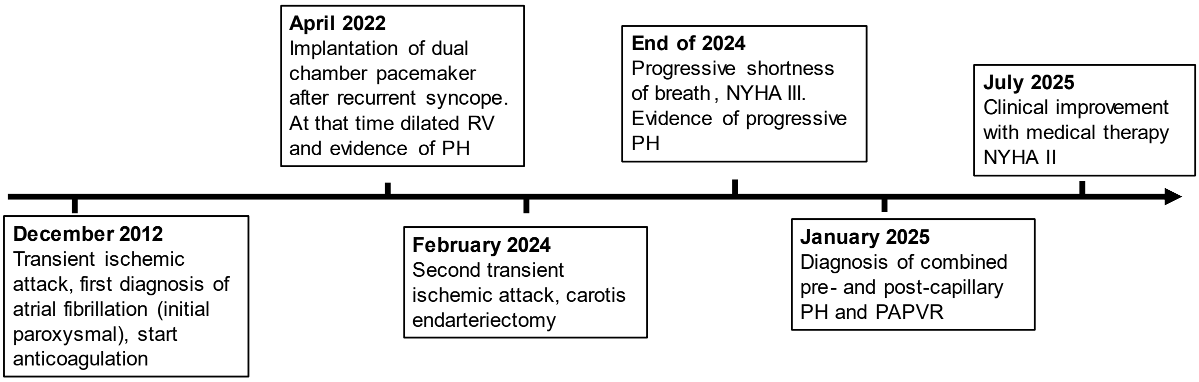

**Legend to videos**

**Video S1.** Transesophageal echocardiography. 4-Chamber mid-esophageal RV view demonstrating a dilated right ventricle with severe tricuspid regurgitation.

**Video S2.** Transesophageal echocardiography. Transgastric view demonstrating severe commissural tricuspid regurgitation.

**Video S3.** 3D Transesophageal echocardiography showing absence of relevant leaflet impingement by the pacemaker lead.
